# Can biological markers improve the management of breast cancer patients?

**DOI:** 10.1054/bjoc.2000.1159

**Published:** 2000-05

**Authors:** Written on behalf of the Biomarkers Ad-hoc Group of the United Kingdom Coordinating Committee on Cancer Research


*British Journal of Cancer* (2000) **82**(10), 1625–1626
© 2000 Cancer Research Campaign
DOI: 10.1054/ bjoc.2000.1159, available online at http://www.idealibrary.com on IDEAL®

### Can biological markers improve the management of breast cancer patients?

**Written on behalf of the Biomarkers Ad-hoc Group of the United Kingdom Coordinating Committee on Cancer Research**

Ideally, biological markers of an individual breast cancer will provide information about (1) prognosis for the patient, (2) the best first-line therapy, (3) response to that therapy, (4) early relapse, (5) targets for new therapies and (6) the biology of breast cancer. No single marker could provide all this information and, in any case, the heterogeneous nature of individual breast cancers makes it unlikely that a single marker could reflect the overall potential of the tumour. This suggests that we should look for groups of markers but so many possible markers have been described over the past 20 years that selection of the best combination of markers will be difficult.

The best established marker in breast cancer is the oestrogen receptor (ER). Its role in prognosis and in predicting response to both adjuvant therapy and therapy for advanced disease has been well described (Elledge and Osborne, 1997; Leake, 1997; EBTG, 1998). Successful quality assurance (QA) exists for the biochemical assay (Romain et al, 1995) and a similar QA scheme is being established for the immunohistochemistry (Barnes et al, 1998) of ER. Largely because of this solid background, a recent study in the UK by Mander et al (1998) showed that only 12.5% of breast cancer units 'do not believe ER status to be important in the management of primary breast cancer' and ER is routinely measured in over 84% of breast disease specialist centres. The authors conclude that failure to measure informative biological markers can 'contribute to differences in outcome between units'. Thus, we have an informative marker, which is both relatively cheap and easy to measure, has a well established QA scheme and is very well defined from studies on large numbers of patients (Romain et al, 1995). However, it has taken 25 years to get routine ER measurement established. If there is a valid case for the use of other markers, as we believe, then we hope that it will not take so long for them to reach routine use!

There is a wide range of potential markers, including oncogene products such as *erb*B-2, anti-oncogene products such as p53, markers of apoptosis/entry into S phase such as bcl-2, markers of cell growth such as Ki-67 and markers of invasion/early relapse such as the urokinase family, whose potential roles have been regularly reported (see Dowsett (1998) for review and Schmitt et al (1997) for details of the potential of the urokinase family). The problem is to select those combinations of biological markers which provide most information for appropriate sub-groups of patients. However, these combinations of markers will only be established in sound manner if each appropriate clinical trial includes a biomarkers component. All protocols proposed through the EORTC are now checked for the presence or possible insertion of such biological end points. Any biomarker study must, of course, obey established rules on tissue handling, assay procedures and data analysis. Appropriate external QA must be included for all multi-centre assays. Hayes et al (1996) have provided one framework to evaluate the clinical utility of such markers.

New technologies such as reverse transcription polymerase chain reaction on fine-needle aspirates, or DNA microarrays make it much more realistic to work on the small quantities of tissue now available. If the result of the application to patient management of specific packages of biomarkers is to improve therapy selection, reduce overtreatment and increase the quality of life, then such an approach will be a cost-effective development.

Is there any evidence that biological markers could act in such a useful manner? Stal et al (1995) carried out a retrospective study of two biomarkers (*erb*B-2 and S Phase Fraction) in patients who had been randomized to receive either radiotherapy or CMF. Patients with high S Phase but low *erb*B-2 responded well to chemotherapy and had a much longer disease-free interval than those treated with radiotherapy, whereas patients with high *erb*B-2 but low S Phase did much better after radiotherapy than after chemotherapy. These results suggest that biological markers can make a difference. Several other retrospective studies will be published shortly. The outcome of prospective studies is eagerly awaited!

### REFERENCES

Barnes DM, Millis RR, Beex LVAM, Thorpe SM and Leake RE (1998) Increased use of immunohistochemistry for oestrogen receptor measurement in mammary carcinoma: the need for quality assurance. *Eur J Cancer* **34:** 1677–1682

Early Breast Cancer Trialists Collaborative Group (1998) Tamoxifen for early breast cancer: an overview of the randomised trials. *Lancet* **351:** 1451–1467

Elledge ME and Osborne CK (1997) Oestrogen receptors and breast cancer. *Br Med J* **314:** 1843–1844

Hayes DF, Bast RC, Desch CF et al (1996) Tumour marker utility grading system: a framework to evaluate clinical utility of tumour markers. *J Natl Cancer Inst* **88:** 1456–1466

Leake RE (1997) Prediction of hormone sensitivity – the receptor years and onwards. *Endocrine-related Cancer* **4:** 289–296

*Correspondence to:* R Leake, Division of Biochemistry and Molecular Biology, Davidson Building, Institute of Biomedical & Life Sciences, University of Glasgow, Glasgow G12 8QQ, UK

Membership of the Biomarkers Group comprises: Professor Robin Leake (Biochemistry, Glasgow University – Chairman), Dr Liz Anderson (Clinical Research, Christie Hospital, Manchester), Professor Mitch Dowsett (Endocrinology, Royal Marsden Hospital, London), Dr Ray Iles (St Bartholomew's Hospital, London), Professor Rob Nicholson, Tenovus Unit, Pharmacology, Cardiff), Professor John Robertson (Surgery, Nottingham), Dr Rosemary Walker (Pathology, Leicester).

Mander BJ, Heal K, Purushotham AD and Wishart GC (1998) Oestrogen receptor status in management of breast cancer in UK. *Lancet* **352:** 36–37

Romain S, Laine Bidron C, Martin PM and Magdelenat H (1995) Steroid receptor distribution in 47,892 breast cancers. A collaborative study of 7 European laboratories. *Eur J Cancer* **31A:** 411–417

Schmitt M, Harbeck N, Thomssen C et al (1997) Clinical impact of the plasminogen activator system in tumour invasion and metastasis: prognostic relevance and target for therapy. *Thrombos Haemostas* **78:** 285–296

Stal O, Sullivan S, Wingren S et al (1995) c-*erb*B 2 expression and benefit from adjuvant chemotherapy and radiotherapy of breast cancer. *Eur J Cancer* **31A:** 2185–2190

*© 2000 Cancer Research Campaign*
